# Quantification of fluorescence angiography for visceral perfusion assessment: measuring agreement between two software algorithms

**DOI:** 10.1007/s00464-024-10794-y

**Published:** 2024-04-09

**Authors:** D. J. Nijssen, J. J. Joosten, J. Osterkamp, R. M. van den Elzen, D. M. de Bruin, M. B. S. Svendsen, M. W. Dalsgaard, S. S. Gisbertz, R. Hompes, M. P. Achiam, M. I. van Berge Henegouwen

**Affiliations:** 1 Department of Surgery, Amsterdam UMC Location University of Amsterdam, Meibergdreef 9, Amsterdam, The Netherlands; 2 Cancer Center Amsterdam, Imaging and Biomarkers, Amsterdam, The Netherlands; 3 Amsterdam UMC Location University of Amsterdam, Biomedical Engineering and Physics, Meibergdreef 9, Amsterdam, The Netherlands; 4 Department of Surgery and Transplantation, Rigshospitalet, Copenhagen University Hospital, Copenhagen, Denmark; 5 Copenhagen Academy for Medical Education and Simulation, Rigshospitalet, Copenhagen University Hospital, Copenhagen, Denmark; 6 Department of Computer Science, SCIENCE, University of Copenhagen, Copenhagen, Denmark

**Keywords:** Fluorescence angiography (FA), Fluorescence time curves, Indocyanine green (ICG), Anastomotic leakage

## Abstract

**Background:**

Indocyanine green fluorescence angiography (ICG-FA) may reduce perfusion-related complications of gastrointestinal anastomosis. Software implementations for quantifying ICG-FA are emerging to overcome a subjective interpretation of the technology. Comparison between quantification algorithms is needed to judge its external validity. This study aimed to measure the agreement for visceral perfusion assessment between two independently developed quantification software implementations.

**Methods:**

This retrospective cohort analysis included standardized ICG-FA video recordings of patients who underwent esophagectomy with gastric conduit reconstruction between August 2020 until February 2022. Recordings were analyzed by two quantification software implementations: AMS and CPH. The quantitative parameter used to measure visceral perfusion was the *normalized maximum slope* derived from fluorescence time curves. The agreement between AMS and CPH was evaluated in a Bland–Altman analysis. The relation between the intraoperative measurement of perfusion and the incidence of anastomotic leakage was determined for both software implementations.

**Results:**

Seventy pre-anastomosis ICG-FA recordings were included in the study. The Bland–Altman analysis indicated a mean relative difference of + 58.2% in the measurement of the *normalized maximum slope* when comparing the AMS software to CPH. The agreement between AMS and CPH deteriorated as the magnitude of the measured values increased, revealing a proportional (linear) bias (*R*^2^ = 0.512, *p* < 0.001). Neither the AMS nor the CPH measurements of the *normalized maximum slope* held a significant relationship with the occurrence of anastomotic leakage (median of 0.081 versus 0.074, *p* = 0.32 and 0.041 vs 0.042, *p* = 0.51, respectively).

**Conclusion:**

This is the first study to demonstrate technical differences in software implementations that can lead to discrepancies in ICG-FA quantification in human clinical cases. The possible variation among software-based quantification methods should be considered when interpreting studies that report quantitative ICG-FA parameters and derived thresholds, as there may be a limited external validity.

The use of indocyanine green fluorescence angiography (ICG-FA) for assessing visceral perfusion during gastrointestinal anastomosis is increasing, as evidence suggests its use might reduce anastomotic leakage rates [1–5]. The technique is based on the concept of optimizing perfusion, an important modifiable risk factor for leakage, during the anastomotic construction [6]. In particular, assessment of esophageal perfusion during esophagectomy with gastric conduit reconstruction may prove useful due to the high anastomotic leakage rate of these procedures (7–30%) [7]. To avoid subjective interpretation of ICG-FA and to obtain an objective threshold for adequate perfusion, multiple research groups have developed software implementations that derive quantitative parameters correlating with intraoperative perfusion [8–10]. However, significant heterogeneity exists between studies investigating quantitative fluorescence angiography (Q-ICG) in methodology, chosen explorative parameters, clinical settings, and outcomes. Additionally, using a fixed camera device minimizes the influence of distance and angulation. Conversely, a freehand device allows enhanced maneuverability and assessment of diverse regions of interest (ROI), yet the variable optical conditions hinder accurate quantification. Hence, identifying reliable parameters for perfusion assessment to predict anastomotic leakage remains challenging. Multiple Q-ICG parameters may be derived from fluorescence time curves (e.g., t-zero [$t0$]*,* time to peak [$ttp$]*,* slope [$slp$], maximum intensity/peak intensity [${F}_{\text{max}}$], and time to half-maximum intensity [${T}_{1/2{\text{max}}}$]). However, intensity parameters, in particular ${F}_{\text{max}}$, are influenced by clinical and optical conditions such as camera distance and angulation to the chosen ROI. Therefore, using Q-ICG parameters that minimize the influence of these circumstances, such as (*normalized) slope* [9] and ${T}_{1/2{\text{max}}}$ [11, 12], have been recommended [13].

One factor that has been less explored in previous literature is the differences among software implementations of similar algorithms by which Q-ICG parameters are calculated. Multiple commercial and non-commercial algorithms exist, and thus, a comparison of these implementations is needed to evaluate the influence of software programming on the quantification of perfusion. Prior comparison of two software implementations demonstrated significant differences in the measurement of Q-ICG parameters [8]. However, this was evaluated in a porcine model and the performance of distinct software-based quantification has yet to be compared in human clinical cases. In this study, we aim to assess the agreement between two previously used and published non-commercially developed software algorithms by applying them to measure perfusion of the esophagus in patients who underwent esophagectomy with gastric conduit reconstruction. As a secondary objective, we aimed to assess whether a correlation exists between the quantitative perfusion evaluation carried out by the two software algorithms and the incidence of anastomotic leakage.

## Materials and methods

### Study design

This study is a collaborative effort of the Amsterdam UMC Hospital, The Netherlands, and Rigshospitalet, Copenhagen University Hospital, Denmark. A retrospective analysis was performed within a patient cohort that underwent esophagectomy with gastric conduit reconstruction in the Amsterdam UMC hospital from August 2020 until February 2022. The study protocol received approval from the Institutional Review Board of Amsterdam UMC, University of Amsterdam, which confirmed that the Medical Research Involving Human Subjects Act (WMO) was not applicable to the study. Written informed consent for data collection and analysis of ICG-FA recordings was obtained from each patient.

Included patients underwent a 2-stage (Ivor Lewis) procedure and were aged ≥ 18 years. We excluded McKeown procedures and recordings of post-anastomosis ICG-FA assessment from our analysis. Additionally, we excluded recordings that had excessive motion artifacts, causing the ROI to move toward other anatomical structures.

### Medical therapy and surgical procedure

Patients received standard neoadjuvant treatment consisting of chemoradiotherapy or perioperative chemotherapy. The Ivor Lewis esophagectomy involves a mobilization of the esophagus, removal of thoracic and abdominal lymph nodes, ligation of key arteries, and eventually the construction of the gastric tube measuring 3–4 cm in width. Subsequently, an intrathoracic stapled anastomosis between the gastric tube and proximal esophagus is created to finalize the reconstruction. More elaborate surgical details were previously reported [14, 15].

### ICG-FA standardization

After the gastric conduit is moved into the thorax, ICG-FA assessment was performed before the creation of the anastomosis. The ROI was predetermined at the planned anastomotic site and was based on visual inspection and the required length of the gastric conduit.

The institutional protocol for standardized ICG-FA assessment involved the following steps:

1. The camera system, PINPOINT (Stryker, Kalamazoo, MI, USA), was fixed using a laparoscope holder at 9 cm distance from the planned anastomotic site.
2. Theater light sources are eliminated except for green light at minimal intensity.
3. A weight-based ICG dosing (0.05 mg/kg/bolus) is administered through a peripheral intravenous line followed by a 10 cc flush of sterile water.
4. The images were recorded for a minimum of 200 s to ensure capturing the outflow phase.

### Quantification of ICG-FA

**Fig. 1:**
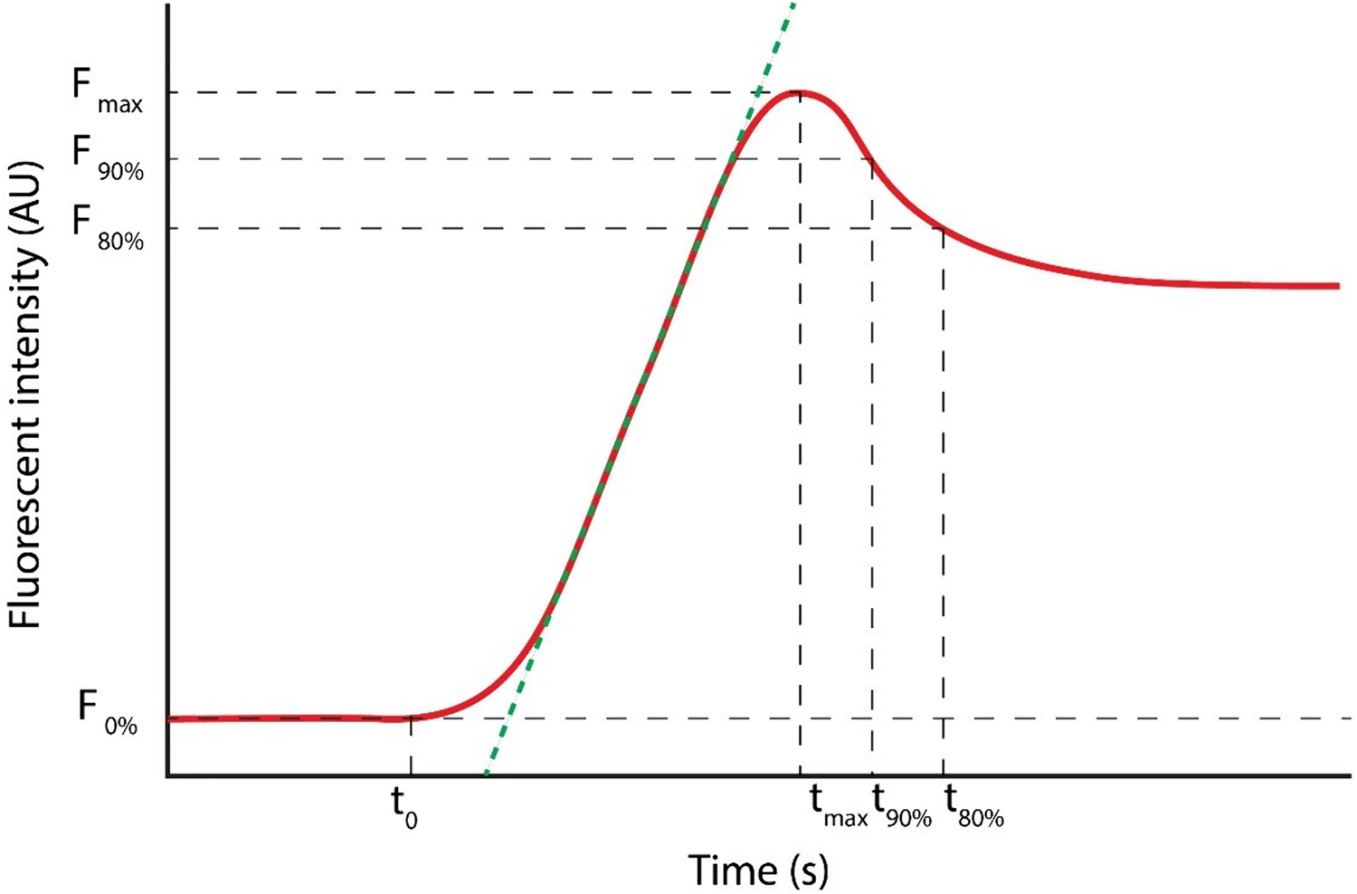
A fluorescence time curve (FTC) and corresponding quantitative parameters; $${F}_{0\%}; {F}_{80\%}$$; $${F}_{90\%}$$; $${F}_{\text{max}}$$: fluorescence intensity in arbitrary units (AU) at minimum, 80% 90% and maximum intensity, respectively. $${t}_{0}$$ here is defined as the time point with the first significantly higher intensity compared to baseline. The green dotted line represents the mean slope (AU/s) of the inflow phase. $${t}_{\text{max}}$$;$${t}_{90\%}$$;$${t}_{80\%}$$ represent the time points at maximum intensity, and after diminishing to 90% and 80% of maximum intensity. The time to peak intensity ($$ttp)$$ is given by $${t}_{\text{max}}- {t}_{0}$$. *Please note that this figure has been previously made by our group and has been published in Surgical Endoscopy by J.J. Joosten et al. *(https://doi.org/10.1007/s00464-023-10107-9) (colour figure online)

ICG-FA recordings were analyzed postoperatively using two independent and non-commercially developed software algorithms (CPH and AMS). The implementations of both quantification methods have been previously reported: AMS [14, 16, 17] and CPH [18–21]. The analysis generated fluorescence time curve (FTC)-derived parameters as illustrated in Fig. 1.

Both quantification algorithms were custom build software implementations written in Python programming language (Python Software Foundation, https://www.python.org/, accessed on 25 April 2022) based on a gray-scale pixel intensity analysis. In the software environments, after choosing a ROI, the software extracts the FTC for that selected ROI and computes the quantitative parameters: *normalized maximum slope, t*_0_*, F*_max_*, tF*_max_*, mean slope, and time to peak *(*ttp*)*.* The raw video data in the AMS software are smoothed using a fourth-order low-pass Butterworth filter with a cutoff frequency of 0.1 Hz, after which the FTC parameters are derived from the smoothed curve. The CPH software uses Lanczos smoothing, which averages neighboring data points throughout the video recording.

### Outcomes and definitions

We predefined the variable *normalized maximum slope* as the primary outcome measure for visceral perfusion based on the previous reports [9, 13]. The software comparison was based on this outcome parameter. Secondary outcomes were time to peak intensity ($$ttp)$$ and the incidence of anastomotic leakage within 90 days postoperatively. Anastomotic leakage was defined in agreement with the Esophagectomy Complications Consensus Group (ECCG) classification [22]. The institutional protocol for the detection of anastomotic leakage involves routine CRP measurement on postoperative days 2 and 3, and on clinical or biochemical indication, a CT scan or endoscopic evaluation. Final confirmation of leakage is established by CT scan, endoscopy, or during reoperation.

### Deriving the normalized maximum slope

The first step toward obtaining the value of the *normalized maximum slope* is the determination of the *maximum slope*. The *maximum slope* is given by the maximum inclination of intensity between two time points. If we define the fluorescence intensity at time $t$ as $F\left(t\right)$ and $\Delta t$ represents the time interval between neighboring points, we can find the *maximum slope* by searching the value of $t$ that maximizes the slope by this formula:

$${\text{Maximum}}\,{\text{slope}} = \max \left( {\frac{{\left( {F\left( t \right) - F\left( {t - \Delta t} \right)} \right)}}{\Delta t}} \right)$$

This value is subsequently normalized by dividing it by the maximum intensity minus the intensity at baseline: ${F}_{\text{max}}-{F}_{0}$

$${\text{Normalized}}\,{\text{maximum}}\,{\text{slope}} = \frac{{{\text{Maximum}}\,{\text{slope}}}}{{F_{\max } - F_{0} }}$$

The *maximum slope* in the AMS software is calculated by finding the single highest value in the derivative of the filtered signal during the entire length of the recording after which it is normalized. The CPH software derives the value for the *maximum slope* from a selected *window of interest* during the inflow phase, with a window size of 50 frames, 25 on each side of the point of maximum slope. After finding the *maximum slope*, normalization is performed following the above formula.

### Data handling

**Fig. 2:**
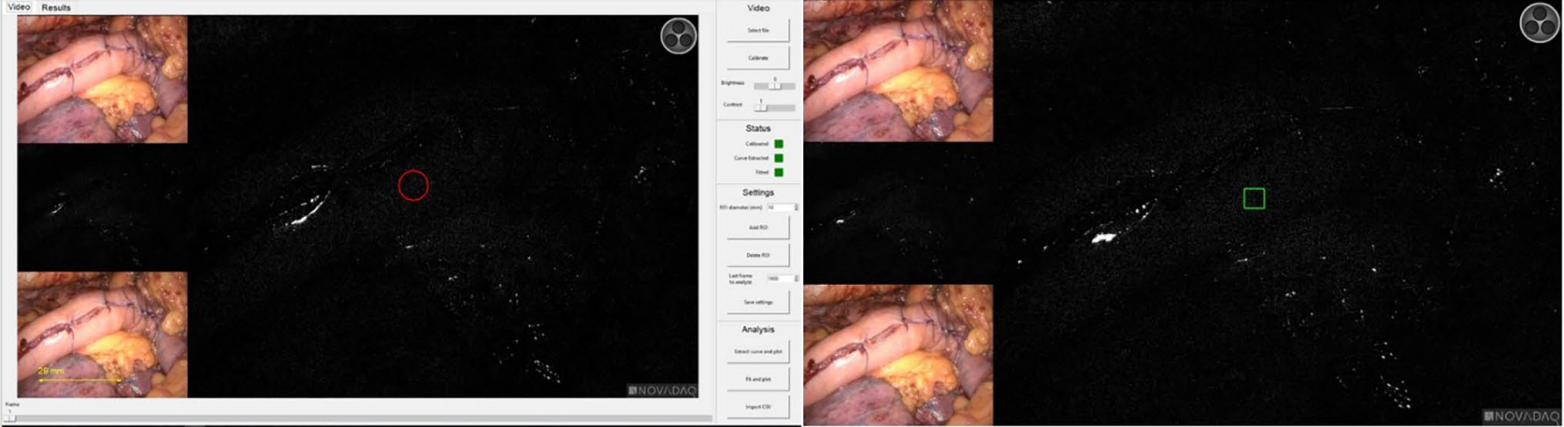
The working environment of both software implementations is displayed, with the AMS (left) and CPH (right) performing analysis of the identical region of interest (ROI) in the same clinical case. ROIs were shared and manually placed during the exact same frame (time point) based on screenshots of the AMS analysis

The anonymized video recordings were shared with the research group based in Copenhagen. In order to obtain comparable outcomes, ROIs defined in Amsterdam were shared with Copenhagen using software screenshots for each video (Fig. 2). The quantitative parameters from both software algorithms were generated in CSV files after which they were shared and compiled in a central SPSS database stored in Amsterdam to perform the analysis.

### Statistics: measuring agreement

Statistical analysis was performed in Statistical Package for Social Sciences (SPSS) of IBM Statistics, version 26.0. Continuous variables are displayed according to their distribution as either mean ± standard deviation or median (IQR). Categorical variables are displayed in absolute number of cases and percentages. The alpha level for statistical significance was set at 0.05. Perfusion measurements from both software implementations are presented in a scatter plot and linear regression is used to evaluate the strength of relation between the observed measurements. The agreement between two different software algorithms considering measurements of the same variables, in this case FTC parameters, was assessed using Bland–Altman plots [23]. The Bland–Altman method for assessing agreement is to derive information from a plot of the difference between two measurements against their mean value. This way, the deviation from the mean FTC parameter *normalized maximum slope* between the two algorithms for each measurement can be plotted, assuming the true value lies closest to the mean of the two measurement methods. Bland–Altman plots are constructed in two ways: plot of the *absolute* and *relative *(*or percentual*) difference against the mean. The relative difference plot is suggested in addition to the absolute difference plot if there is an increased variability of the differences as the measurement magnitude increases [24]. A one-sample *T*-test is used to calculate the mean difference (*d*) and the standard deviation of the differences ($s$) to express bias and the limits of agreement.The assumption of normal distribution of the continuous measurement variables in the Bland–Altman analysis is tested with a Shapiro–Wilk test.

The outcome of the *normalized maximum slope* measurement by the two software implementations was compared between patients with and without anastomotic leakage using a Mann–Whitney *U*-test.

## Results

**Fig. 3:**
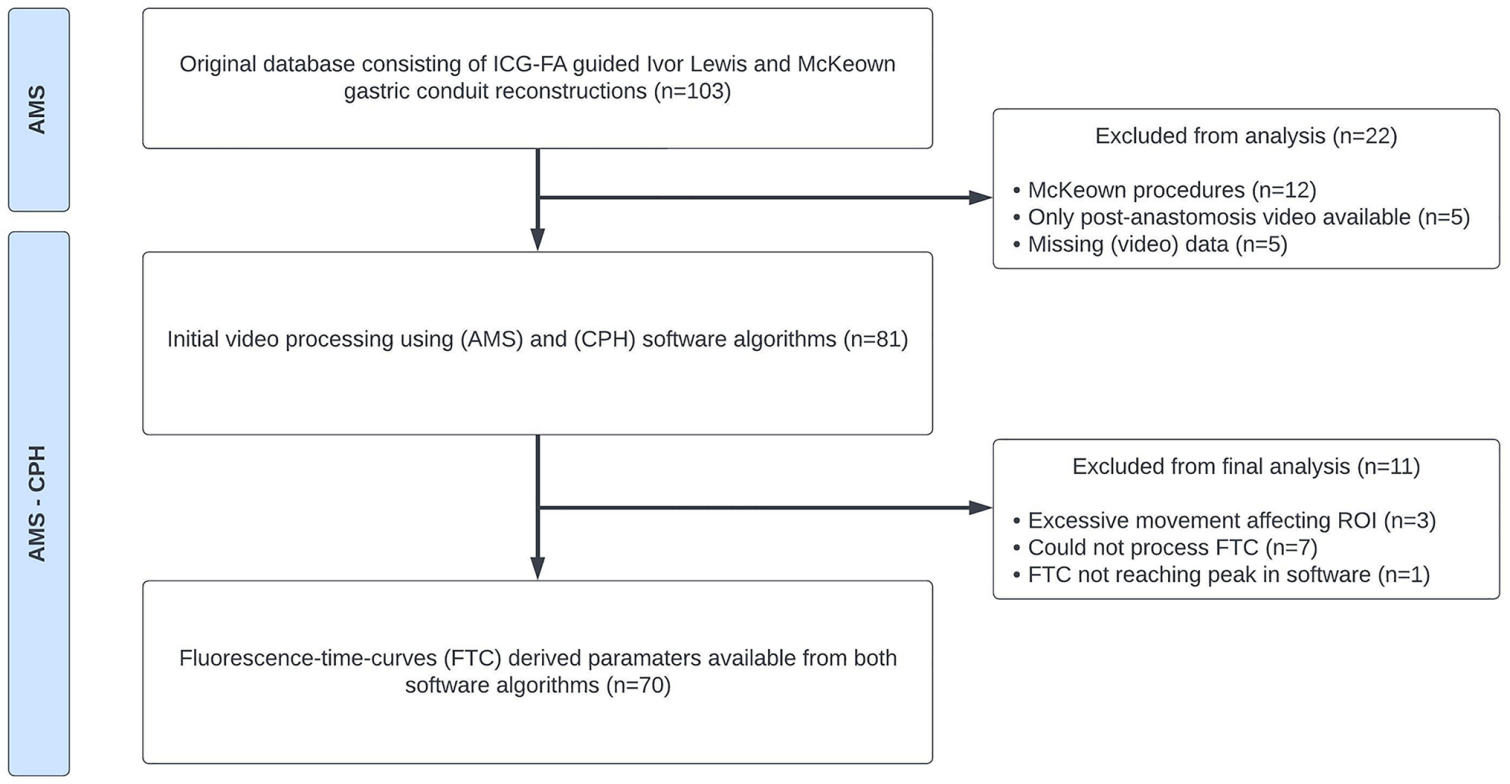
A flowchart depicting the flow of patients from the original dataset (*n* = 103) with reasons for exclusion in the final software comparison

Out of a total of 81 pre-anastomosis video recordings of ICG-FA-guided Ivor Lewis procedures, 70 recordings could be analyzed using both software algorithms and were included in the final analysis. Figure 3 displays a flowchart of included cases and reasons for exclusion.

### Data examination

**Fig. 4:**
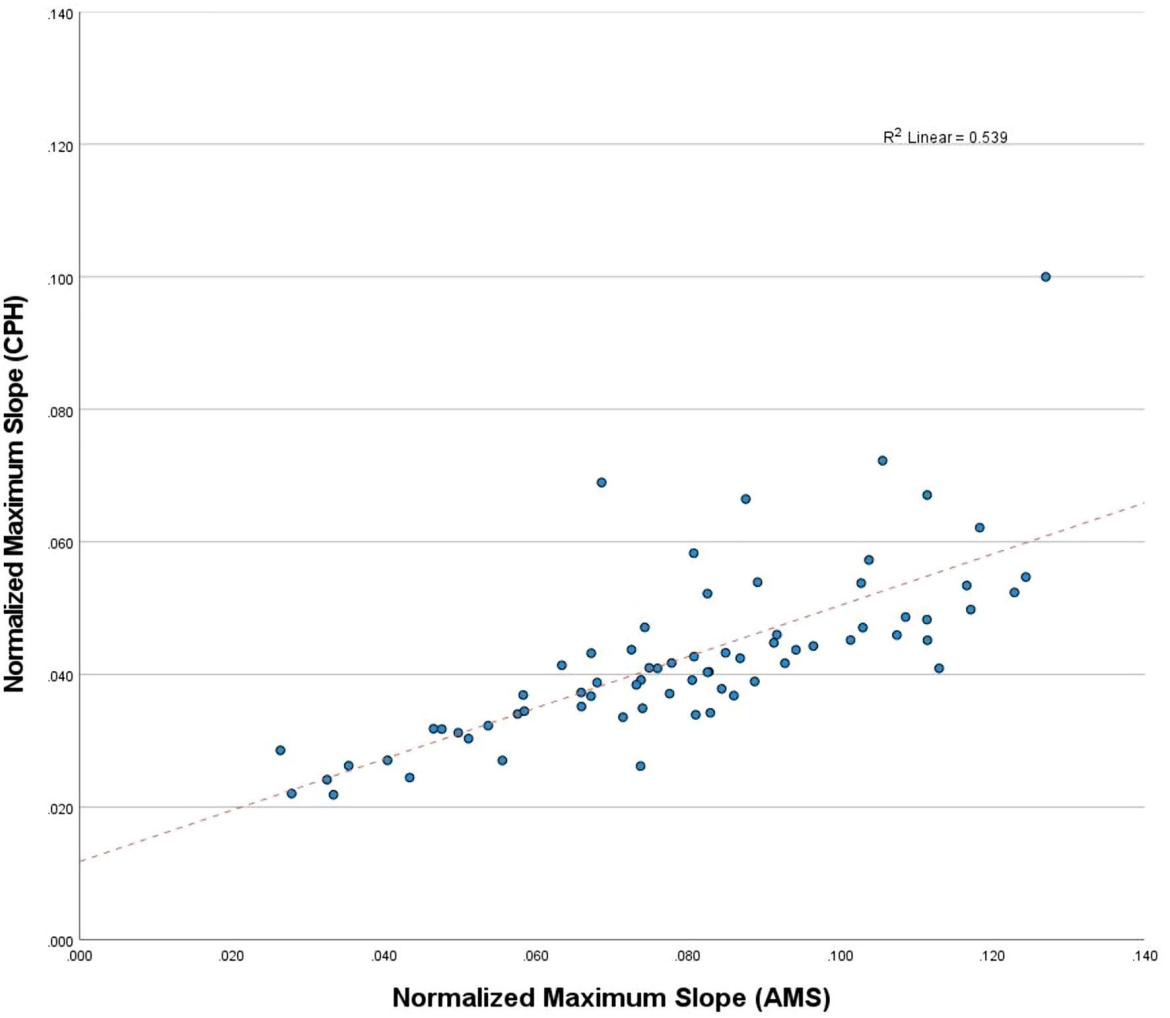
A simple scatter plot of the measured values of the normalized maximum slope by both software implementations (AMS on the *X*-axis and CPH on the *Y*-axis). The red dotted line represents the linear regression function obtained by correlating both measurement methods (*R*^2^: 0.539, *p* < 0.001) (colour figure online)

In Fig. 4, a scatter plot for the primary outcome variable for perfusion assessment, the *normalized maximum slope*, is presented, displaying results from AMS implementation on the *X*-axis and CPH implementation on the *Y*-axis. The assumption of a normal distribution (Gaussian) of the observed measurements was valid (*p* = 0.441 (AMS), *p* = 0.084 (CPH)). The results of the linear regression indicated that 53.9% of the variance in the CPH measurements is explained by the AMS measurements. The fitted regression model was described by $$normalized maximum slope \left(AMS\right)=0.020+1.396*normalized maximum slope \left(CPH\right)$$ and was statistically significant (*R*^2^ = 0.539,* F*(0.023, 0.020) = 80.83,* p* < 0.001).

**Fig. 5:**
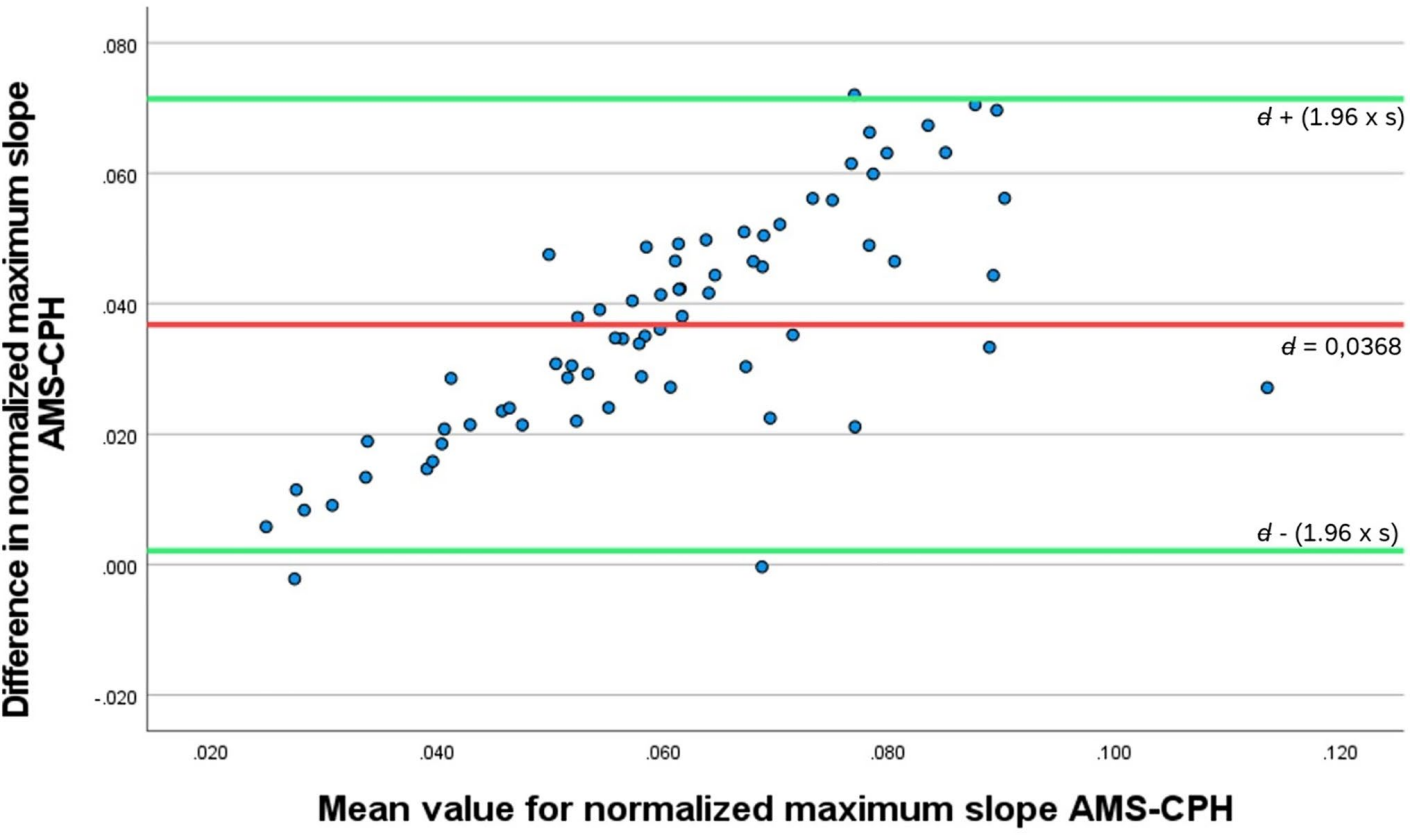
A Bland–Altman plot of the differences between the AMC and CPH measurements (*Y*-axis) and the mean value of the two measurements (*X*-axis). The red line represents the mean difference *d* of 0.037 s^−1^ higher output for the *normalized maximum slope* computed by the AMS software compared to the CPH software. The green lines represent the lower and upper limit of agreement (LoA), given by *d* ± 1.96 × standard deviation of the differences (s): 0.002 − 0.071 s^−1^, respectively (colour figure online)

Figure 5 displays the plot of the differences between the AMS and CPH measurements against their mean value (Bland–Altman plot). The red line on the *X*-axis represents the mean difference (*d*) between the two software measurement methods, which is 0.037 s^−1^ for the *normalized maximum slope,* meaning that on average, the output from the AMS software is 0.037 s^−1^ higher than from the CPH software. The differences between each software measurement for the *normalized maximum slope* were significantly different from zero (*p* <0.001); thus, inherent difference between two software calculations is indicated. Accordingly, if the two software measurements were in perfect agreement, the mean difference would be equal or consistently close to zero (*=* *no difference*). The green lines represent the *limits of agreement*, established by the 95% confidence interval surrounding (± (1.96 × $s$)): (0.002 − 0.071 s^−1^).

From the Bland–Altman plot of the *normalized maximum slope,* we determined visually a proportional bias between the two measurement methods. The linear regression model indicated a significant relationship between the mean measurements and the differences (*R*^2^ = 0.512, *F*(0.011, 0.011) = 71.29,* p* < 0.001). The resulting model demonstrated a positive linear relationship following the function ${\text{difference}}\left({\text{AMS}}-{\text{CPH}}\right)=-0.006+0.708*{\text{mean}} \text{(}\frac{\text{AMS+CPH}}{2}\text{)}$. Accordingly, the differences between the two measurement methods become proportionally larger with higher mean values.

**Fig. 6:**
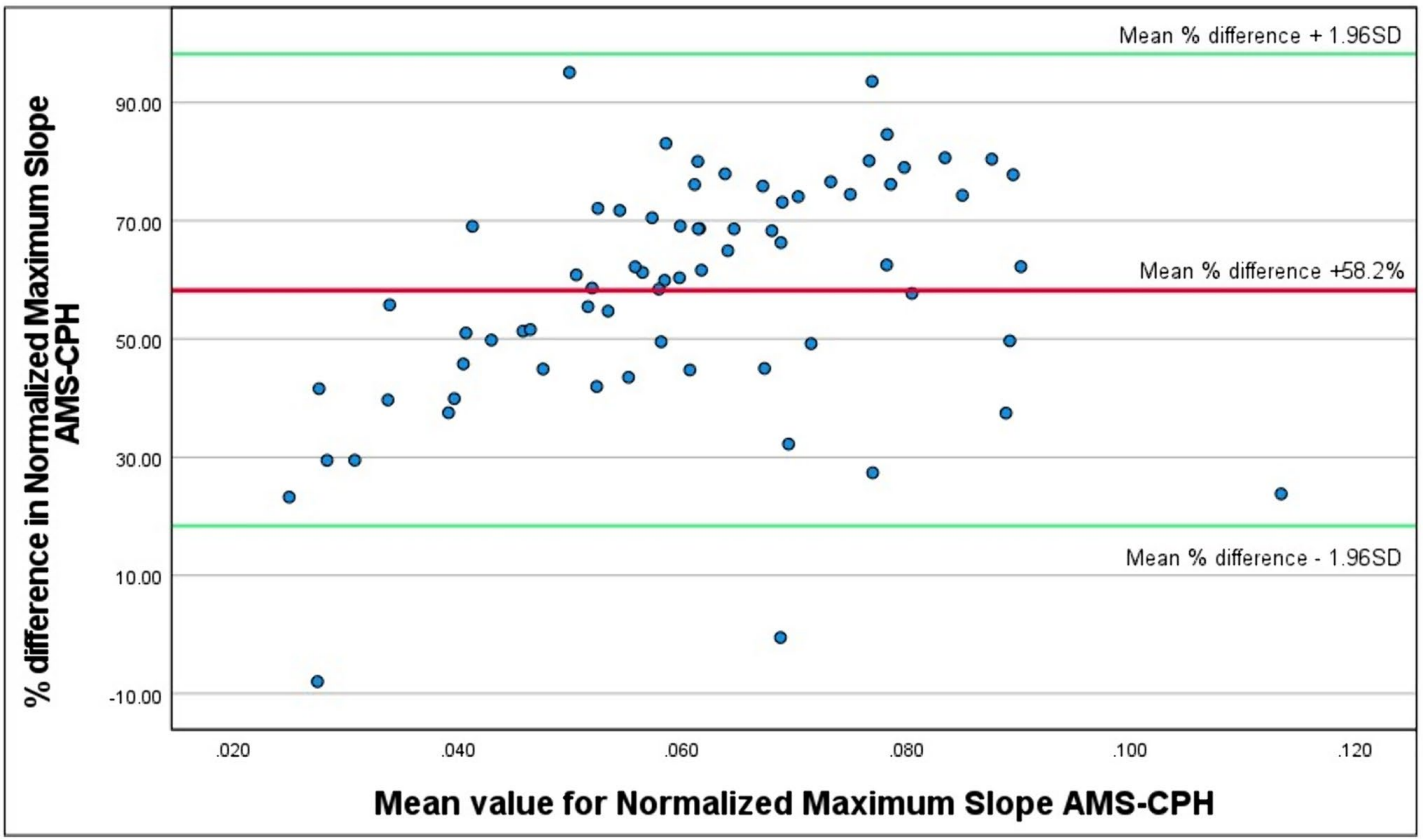
A relative difference Bland–Altman plot depicting the difference as percentage of deviation (*Y*-axis) between the two software methods (i.e., [(value AMS – CPH)/mean × 100%] against the mean (*X*-axis). The red line represents the mean difference expressed as percentage; on average, a +58.2% higher value is computed by the AMS software compared to the CPH software. The green lines represent the lower and upper Limit of Agreement (LoA): + 18.4% − + 98.3%, respectively (colour figure online)

A Bland–Altman plot of the relative difference between each measurement against the mean value of both measurements is displayed in Fig. 6*.* A bias (mean difference) of +58.2% is observed from the plot. The limits of agreement (mean % difference ± (1.96 × $s$)) in the plot ranged from + 18,4% to + 98,26%. Visually, a trend toward linear bias remained; however, this observation was less pronounced in the relative difference plot compared to the absolute difference plot in Fig. 5 (*R*^2^ = 0.130,* F*(3597.307, 24140.007) = 10.133,* p* < 0.002). The fitted linear model is described by % difference = 33.551 + 404.166*$\text{mean (}\frac{\text{AMS+CPH}}{2}\text{)}$.

**Fig. 7:**
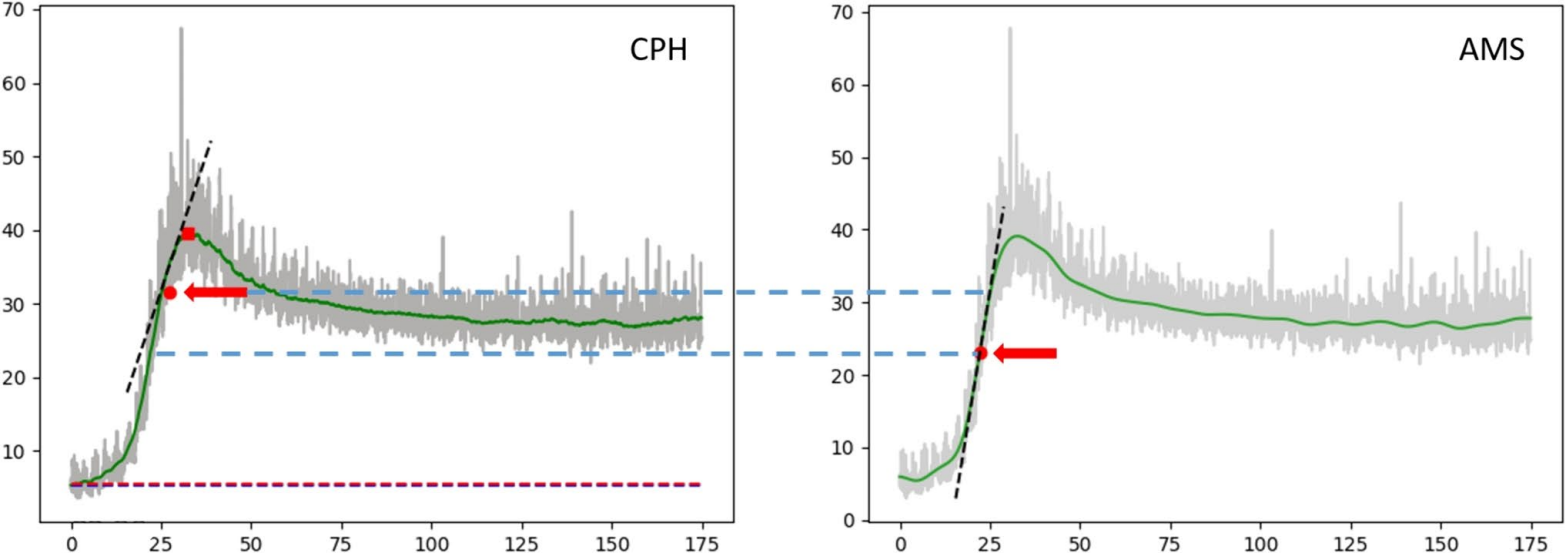
Analysis of an ICG-FA assessment by the CPH and AMS software. The red arrow indicates the *maximum slope* or maximum inclination of intensity per frame [${\text{max}}$($\Delta F/\Delta t$)] and the blue dotted line displays how it relates to the other software computation of the *maximum slope*. The left CPH analysis retrieves a less steep *maximum slope* compared to the AMS analysis on the right. This effect is caused by two main components: firstly, CPH calculates a regression over a larger time window of interest, 25 frames on each side of [${\text{max}}$($\Delta F/\Delta t$)], whereas AMS uses just [${\text{max}}$($\Delta F/\Delta t$)]. This will make the CPH measurement always lower as it includes points below [${\text{max}}$($\Delta F/\Delta t$)], which also causes the measurements to diverge more with higher slopes. This is also indicated by the proportional bias in the Bland–Altman plot (Fig. 5 and 6). Secondly, the difference in filtering can influence the position of the *maximum slope*. Notice that the filtering of AMS generates a smoother FTC, in which steeper components of the raw signal (here in gray) are filtered out, thus generating a different position of the *maximum slope* than in the relatively less filtered CPH generated FTC. Green line—*smoothed intensity.* Gray line—*Raw video signal* (colour figure online)

Figure 7 portrays the software specific translation of the raw video data into a smoothed function representing the FTC from which the quantitative parameters are derived. The figure illustrates how the selection of a window of interest for calculating the *normalized maximum slope* influences the magnitude of the value and how filtering can cause the positioning of the maximum slope (and window of interest in CPH) to diverge between the two methods.

**Fig. 8:**
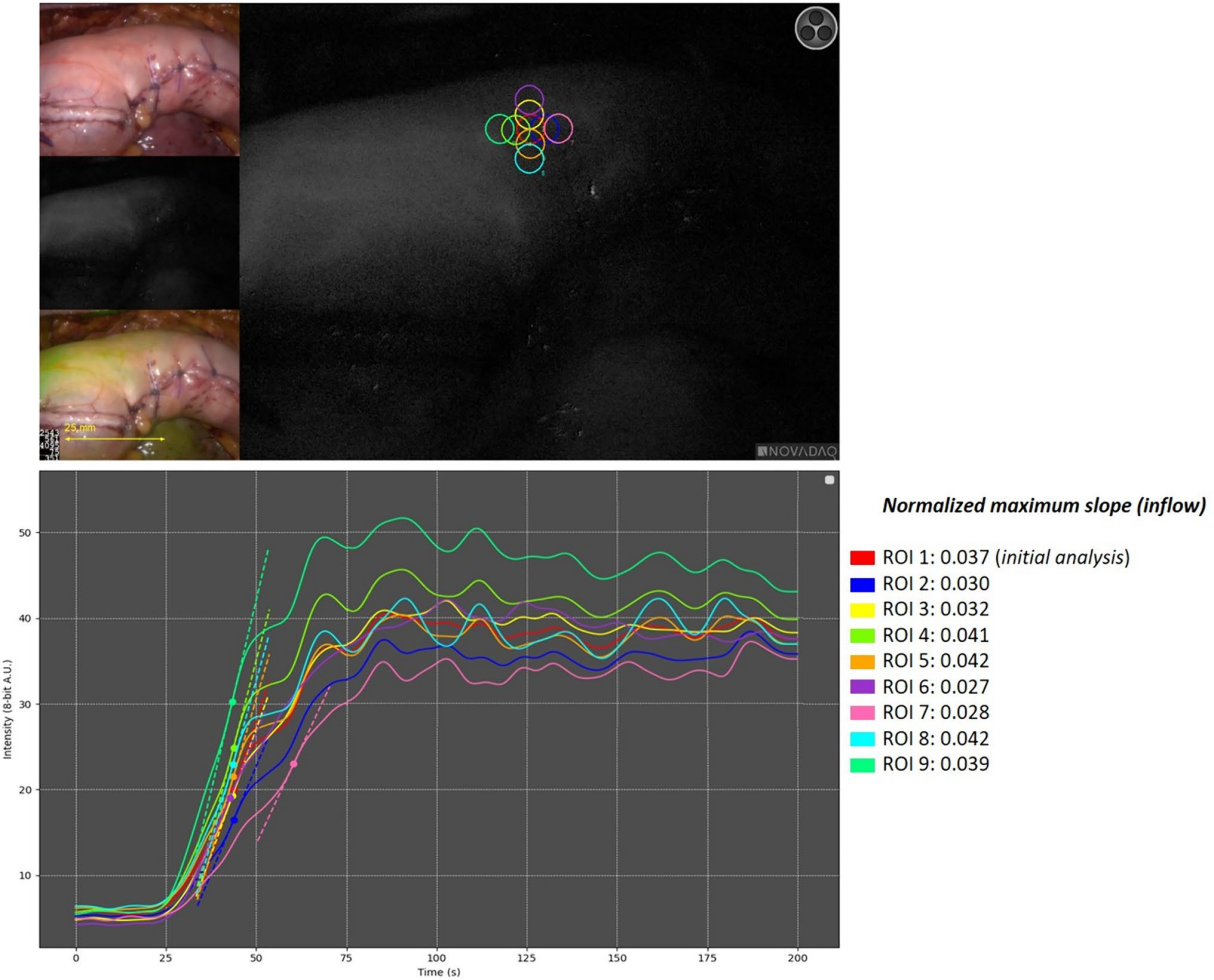
The figure displays an ICG-FA assessment in which 8 alternative ROIs are manually placed in close proximity of the original ROI 1 in the analysis by the AMS software. The four “inner” ROI’s (ROI 2, ROI 3, ROI 4, and ROI 5) are positioned in closest proximity to the original ROI 1, representing displacement as could occur by inter-user variation. The “outer” four ROIs (ROI 6, ROI 7, ROI 8, and ROI 9) illustrate how the *normalized maximum slope* further deviates from the original ROI 1 when moved along the gastric conduit. The effect on the computation of the *normalized maximum slope* relative to each ROI is shown on the right. The obtained values for the inner ROIs ranged from 0.030 to 0.042 compared to the original ROI 1 measuring 0.037 (− 18.9% to + 13.5%, respectively).

In Fig. 8, the influence of manual ROI placement on the resulting *normalized maximum slope* value is examined. The displacement of the four inner ROIs in closest proximity to the original ROI resulted in measurements ranging from 0.030 to 0.042 compared to the original ROI measuring 0.037 (− 18.9% to + 13.5%, respectively).

### Clinical outcomes

Table 1 displays the clinical outcomes of the included patients. Anastomotic leakage occurred in 9 out of 70 (12.9%) patients. The *normalized maximum slope* in the anastomotic leakage group was slightly less steep in the AMS software; in the CPH software, the values were nearly identical (median of 0.081 versus 0.074, *p *= 0.32 and 0.041 vs 0.042, *p *= 0.51, respectively). None of these values approached statistical significance at *alpha* = 0.05.

**Table 1:** Normalized maximum slope measurement by the occurrence of anastomotic leakage

Parameter	No anastomotic leakage		Anastomotic leakage		*p* value*	
	AMS	CPH	AMS	CPH	AMS	CPH
Normalized maximum slope	0.081 (0.067–0.010)	0.041 (0.035–0.048)	0.074 (0.057–0.089)	0.042 (0.031–0.045)	0.32	0.51

Values are in median (IQR)

*Mann–Whitney *U*-test

## Discussion

This study is the first to investigate the agreement between two independently developed software algorithms for calculating quantitative ICG-FA parameters in a clinical dataset of human subjects. Our results show that, despite the correlated outcomes between the two software algorithms, there is substantial variability in the derived measurements from the FTCs. Furthermore, our findings indicate a linear bias in the absolute values between both software algorithms, causing the differences in methods to increase with higher *normalized maximum slope* values*.*

The quantification of ICG-FA for assessing gastrointestinal perfusion has been a particular topic of interest because compromised tissue perfusion is considered an important modifiable risk factor for anastomotic leakage [25–28]. While subjective assessment of ICG-FA effectively prevents surgical morbidity in gastrointestinal anastomoses [4], the technique’s considerable learning curve and susceptibility to variable interpretation must be considered [29]. Therefore, objective and reliable quantification methods are essential for broader effective clinical adoption. However, the widespread clinical adoption of quantitative fluorescence assessment faces several challenges, including the lack of calibration standards, standardization in technical specifications of available imaging systems, software algorithms, and protocols across institutions, which inherently limit the external validity of established quantification techniques. Transparent reporting of technical details and specific use requirements for quantification methods is crucial for comprehending how different approaches can be applied across diverse clinical settings. This can significantly amplify the practical implications of study results, extending their utility beyond the original research context.

A previous report assessed the comparability of two independently developed software-based quantification methods for ICG-FA in a standardized porcine dataset [8]. The results indicated a significant difference in quantitative ICG-FA measurements derived from the software algorithms in low- and high-perfusion areas. While our results align with these findings, the two quantification methods discussed in the referenced study were compared in a porcine model with a surgical procedure performed in the small intestine. In addition to a distinct clinical setting, the referenced comparison was based on the correlation between the *normalized maximum slope* measurements. In our approach, we treated the software implementations as genuine measurement instruments. This led to the application of a statistical method that produces a different interpretation and presentation of the results. Specifically, our approach displays the agreement for each individual measurement rather than obtaining a correlation between them. While these factors may hinder exact comparison between the two studies, both results point to divergent measurements by different software implementations. In the current study, the quantification of perfusion in both AMS and CPH algorithms is based on the same quantitative fluorescence parameter. The perfusion is assessed by calculating the *normalized maximum slope* derived from the raw ICG-FA video recordings. The maximum inclination of fluorescence intensity between two points in time (*maximum slope*) is calculated and adjusted for the intensity range of the fluorescence signal (*normalization*). While the values for the *normalized maximum slope* generated by the two methods hold moderate correlation, the proportional bias between them implicates that other variables must influence the analysis. Considering the use of an identical dataset, aside from minimal software user influence, any observed difference in the measured parameters must be rooted in software programming approaches [17].

The signal processing from raw video data is one factor that effectuated the difference in outcome. Some form of signal smoothing is needed to obtain the FTC-derived parameters. The AMS software uses a low-pass filter that decreases disparity between nearby data points by attenuating high-frequency components while allowing low-frequency components to pass through. The CPH software uses Lanczos smoothing, which averages neighboring data points throughout the video recording. The CPH approach produced a less smoothed FTC, leaving more of the raw signal intact, with steeper components remaining and influencing the computation of the *maximum slope*. On the contrary, while the AMS approach produces a smoother FTC, this also risks filtering out relevant data points from the raw data. It is unclear whether one filtering approach is superior because the “true” FTC is unknown, leaving no golden reference standard. The development of simulated videos with standardized amounts of intensity and noise (motion) artifacts to emulate reference standard FTCs may hold the potential to aid future validation of different software algorithms. Another variable influencing the divergent findings was the computation method for finding the *normalized maximum slope.* The CPH software determines the regression for the *normalized maximum slope* within a set time window of interest of 50 frames long (approximately 2 s). Alternatively, the value from the AMS software is given by the single highest value in the derivative of the smoothed signal during the entire length of the recording. Because the CPH software computes the *normalized maximum slope* in a window of interest, it inherently results in a lower value as it includes points below [max(∆*F*/∆*t*)]. This accounts for the consistently higher measurements obtained by the AMS software. Inter-user variation cannot be excluded as a possible origin for any differences in outcome, given that the chosen ROI and its size are placed manually for each video analysis. However, this influence is expected to be minimal, given that the differences observed from slight displacement of the ROI led to only marginal variability in obtained measurements.

The scope of this study was limited to comparing a single Q-ICG parameter, as proof of concept that technical differences lead to significant differences in observed parameter values. Relatedly, the present study was not powered to determine a correlation between quantitative measures and clinical outcomes, and no such relationship was observed. However, a range of Q-ICG parameters have shown to correlate to clinical outcomes in previous reports, ranging from a pragmatic cutoff value for time until fluorescence enhancement to more specific parameters as the (normalized) maximum slope, ${T}_{1/2{\text{max}}}$, ${T}_{0}$ or time until maximum enhancement [12, 14, 30–32]. Interestingly, one study distinguished three distinct perfusion patterns rather than focusing on single or combined Q-ICG parameters in a similar study population of patients undergoing an esophagostomy with gastric conduit reconstruction [33]. In that study, groups were too small to determine how these perfusion patterns related to clinical endpoints. The conjunction of multiple Q-ICG parameters translating to a perfusion pattern might be a topic of interest for future studies aiming to strengthen the current technology. Artificial intelligence might form a promising tool for distinct pattern recognition related to clinical endpoints in these datasets, as described for tissue classification of colorectal lesions using ICG-FA [34]. Such advancements in software-based quantification may eventually influence clinical decision making in gastrointestinal surgery, for example, by selecting high- or low-risk patients for perfusion-related complications. This information can potentially be used to optimize and tailor the individual postoperative care pathway.

The current study has certain limitations. We investigated the differences in output for a single Q-ICG parameter using two non-commercially developed software algorithms. Therefore, there is inherently limited generalizability of our results as it does not depict the comparability of other software-based quantification methods. Other quantification methods might use alternative Q-ICG parameters to assess visceral perfusion or predict clinical outcomes, and the comparability of software quantification might differ according to the clinical setting. Secondly, the exclusion of cases due to excessive motion artifacts and processing errors by either of the two software implementations may have led to a selection bias toward higher-quality video recordings. This could have influenced the agreement between the software implementations, as it is expected that the agreement declines in recordings disturbed by motion. Relatedly, it is essential to acknowledge that the present analysis is constrained by the suitability of the dataset. The ICG-FA recordings of the gastric conduit reconstructions are susceptible to respiratory movement and encompass a narrow and difficult-to-reach ROI, which challenges the analysis and potentially introduces greater variability compared to other clinical settings. Future comparisons in other clinical settings may show greater agreement, also considering current advances in software implementations such as motion-tracking algorithms.

In conclusion, our study illustrated the impact of divergent software programming approaches on the outcome of two software-based quantification methods for ICG-FA. Technical differences in software implementations can lead to discrepancies in the measured parameters magnitude. The possible variation among software-based quantification methods should be considered when interpreting studies that report quantitative ICG-FA parameters and derived thresholds. These variations have the potential to limit the external validity of such findings.

## References

[1] Ladak F, Dang JT, Switzer N, Mocanu V, Tian C, Birch D, Turner SR, Karmali S (2019). Indocyanine green for the prevention of anastomotic leaks following esophagectomy: a meta-analysis. Surg Endosc.

[2] Li Z, Zhou Y, Tian G, Liu Y, Jiang Y, Li X, Song M (2021). Meta-analysis on the efficacy of indocyanine green fluorescence angiography for reduction of anastomotic leakage after rectal cancer surgery. Am Surg.

[3] Meyer J, Joshi H, Buchs NC, Ris F, Davies J (2022). Fluorescence angiography likely protects against anastomotic leak in colorectal surgery: a systematic review and meta-analysis of randomised controlled trials. Surg Endosc.

[4] Slooter MD, Eshuis WJ, Cuesta MA, Gisbertz SS, van Berge Henegouwen MI (2019). Fluorescent imaging using indocyanine green during esophagectomy to prevent surgical morbidity: a systematic review and meta-analysis. J Thorac Dis.

[5] Watanabe J, Takemasa I, Kotake M, Noura S, Kimura K, Suwa H, Tei M, Takano Y, Munakata K, Matoba S, Yamagishi S, Yasui M, Kato T, Ishibe A, Shiozawa M, Ishii Y, Yabuno T, Nitta T, Saito S, Saigusa Y, Watanabe M (2023). Blood perfusion assessment by indocyanine green fluorescence imaging for minimally invasive rectal cancer surgery (EssentiAL trial): a randomized clinical trial. Ann Surg.

[6] Zarnescu EC, Zarnescu NO, Costea R (2021). Updates of risk factors for anastomotic leakage after colorectal surgery. Diagnostics (Basel).

[7] Low DE, Kuppusamy MK, Alderson D, Cecconello I, Chang AC, Darling G, Davies A, D’Journo XB, Gisbertz SS, Griffin SM (2019). Benchmarking complications associated with esophagectomy. Ann Surg.

[8] Gosvig K, Jensen SS, Qvist N, Nerup N, Agnus V, Diana M, Ellebæk MB (2021). Quantification of ICG fluorescence for the evaluation of intestinal perfusion: comparison between two software-based algorithms for quantification. Surg Endosc.

[9] Nerup N, Andersen HS, Ambrus R, Strandby RB, Svendsen MBS, Madsen MH, Svendsen LB, Achiam MP (2017). Quantification of fluorescence angiography in a porcine model. Langenbecks Arch Surg.

[10] Vaassen H, Wermelink B, Geelkerken B, Lips D (2022). Fluorescence angiography for peri-operative assessment of bowel viability in patients with mesenteric ischaemia. EJVES Vasc Forum.

[11] Kamiya K, Unno N, Miyazaki S, Sano M, Kikuchi H, Hiramatsu Y, Ohta M, Yamatodani T, Mineta H, Konno H (2015). Quantitative assessment of the free jejunal graft perfusion. J Surg Res.

[12] Son GM, Kwon MS, Kim Y, Kim J, Kim SH, Lee JW (2019). Quantitative analysis of colon perfusion pattern using indocyanine green (ICG) angiography in laparoscopic colorectal surgery. Surg Endosc.

[13] Lütken CD, Achiam MP, Osterkamp J, Svendsen MB, Nerup N (2021). Quantification of fluorescence angiography: toward a reliable intraoperative assessment of tissue perfusion—a narrative review. Langenbecks Arch Surg.

[14] Joosten JJ, Slooter MD, van den Elzen RM, Bloemen PR, Gisbertz SS, Eshuis WJ, Daams F, de Bruin DM, van Berge Henegouwen MI (2023). Perfusion assessment by fluorescence time curves in esophagectomy with gastric conduit reconstruction: a prospective clinical study. Surg Endosc.

[15] Slaman AE, Eshuis WJ, van Berge Henegouwen MI, Gisbertz SS (2023). Improved anastomotic leakage rates after the “flap and wrap” reconstruction in Ivor Lewis esophagectomy for cancer. Dis Esophagus.

[16] Joosten JJ, Slooter MD, van den Elzen RM, Bloemen PR, Laméris W, de Bruin DM, Bemelman WA, Hompes R (2023). Understanding fluorescence time curves during ileal pouch-anal anastomosis with or without vascular ligation. Surg Endosc.

[17] Joosten JJ, Bloemen PR, van den Elzen RM, Dalli J, Cahill RA, van Berge Henegouwen MI, Hompes R, de Bruin DM (2023). Investigating and compensating for periphery-center effect among commercial near infrared imaging systems using an indocyanine green phantom. Appl Sci.

[18] Nerup N, Svendsen MBS, Rønn JH, Konge L, Svendsen LB, Achiam MP (2022). Quantitative fluorescence angiography aids novice and experienced surgeons in performing intestinal resection in well-perfused tissue. Surg Endosc.

[19] Osterkamp J, Strandby R, Nerup N, Svendsen M, Svendsen L, Achiam M (2021). Quantitative fluorescence angiography detects dynamic changes in gastric perfusion. Surg Endosc.

[20] Nerup N, Knudsen KBK, Ambrus R, Svendsen MBS, Thymann T, Ifaoui IBR, Svendsen LB, Achiam MP (2017). Reproducibility and reliability of repeated quantitative fluorescence angiography. Surg Technol Int.

[21] Nerup N, Andersen HS, Ambrus R, Strandby RB, Svendsen MBS, Madsen MH, Svendsen LB, Achiam MP (2017). Quantification of fluorescence angiography in a porcine model. Langenbeck's Arch Surg.

[22] Low DE, Alderson D, Cecconello I, Chang AC, Darling GE, D’Journo XB, Griffin SM, Hölscher AH, Hofstetter WL, Jobe BA, Kitagawa Y, Kucharczuk JC, Law SY, Lerut TE, Maynard N, Pera M, Peters JH, Pramesh CS, Reynolds JV, Smithers BM, van Lanschot JJ (2015). International consensus on standardization of data collection for complications associated with esophagectomy: Esophagectomy Complications Consensus Group (ECCG). Ann Surg.

[23] Bland JM, Altman D (1986). Statistical methods for assessing agreement between two methods of clinical measurement. Lancet.

[24] Giavarina D (2015). Understanding Bland Altman analysis. Biochem Med (Zagreb).

[25] Ikeda Y, Niimi M, Kan S, Shatari T, Takami H, Kodaira S (2001). Clinical significance of tissue blood flow during esophagectomy by laser Doppler flowmetry. J Thorac Cardiovasc Surg.

[26] van Rooijen SJ, Huisman D, Stuijvenberg M, Stens J, Roumen RMH, Daams F, Slooter GD (2016). Intraoperative modifiable risk factors of colorectal anastomotic leakage: why surgeons and anesthesiologists should act together. Int J Surg.

[27] Fabbi M, Hagens ERC, van Berge Henegouwen MI, Gisbertz SS (2020). Anastomotic leakage after esophagectomy for esophageal cancer: definitions, diagnostics, and treatment. Dis Esophagus.

[28] Zehetner J, DeMeester SR, Alicuben ET, Oh DS, Lipham JC, Hagen JA, DeMeester TR (2015). Intraoperative assessment of perfusion of the gastric graft and correlation with anastomotic leaks after esophagectomy. Ann Surg.

[29] Hardy NP, Dalli J, Khan MF, Andrejevic P, Neary PM, Cahill RA (2021). Inter-user variation in the interpretation of near infrared perfusion imaging using indocyanine green in colorectal surgery. Surg Endosc.

[30] Slooter MD, de Bruin DM, Eshuis WJ, Veelo DP, van Dieren S, Gisbertz SS, van Berge Henegouwen MI (2021). Quantitative fluorescence-guided perfusion assessment of the gastric conduit to predict anastomotic complications after esophagectomy. Dis Esophagus.

[31] Iwamoto H, Matsuda K, Hayami S, Tamura K, Mitani Y, Mizumoto Y, Nakamura Y, Murakami D, Ueno M, Yokoyama S, Hotta T, Takifuji K, Yamaue H (2020). Quantitative indocyanine green fluorescence imaging used to predict anastomotic leakage focused on rectal stump during laparoscopic anterior resection. J Laparoendosc Adv Surg Tech A.

[32] Bach Korsholm Knudsen K, Nerup N, Thorup J, Thymann T, Sangild PT, Svendsen LB, Achiam M, Svendsen MBS, Lauritsen T, Leth Maroun L, Ifaoui IBR (2022). Intestinal perfusion assessed by quantitative fluorescence angiography in piglets with necrotizing enterocolitis. J Pediatr Surg.

[33] Galema HA, Faber RA, Tange FP, Hilling DE, van der Vorst JR, de Steur WO, Hartgrink HH, Vahrmeijer AL, Hutteman M, Mieog JSD, Lagarde SM, van der Sluis PC, Wijnhoven BPL, Verhoef C, Burggraaf J, Keereweer S (2023). A quantitative assessment of perfusion of the gastric conduit after oesophagectomy using near-infrared fluorescence with indocyanine green. Eur J Surg Oncol.

[34] Hardy NP, Mac Aonghusa P, Neary PM, Cahill RA (2021). Intraprocedural artificial intelligence for colorectal cancer detection and characterisation in endoscopy and laparoscopy. Surg Innov.

